# The Prognosis of Nasopharyngeal Carcinoma Involving Masticatory Muscles: A Retrospective Analysis for Revising T Subclassifications: Erratum

**DOI:** 10.1097/01.md.0000471263.32665.89

**Published:** 2015-08-28

In the article “The Prognosis of Nasopharyngeal Carcinoma Involving Masticatory Muscles: A Retrospective Analysis for Revising T Subclassifications”,^1^ which appeared in Volume 94, Issue 4 of *Medicine*, the “With” and “Without” curves of Figure 2 were mislabeled. The article has since been corrected online.
